# Quality Improvement to Increase Breastfeeding in Preterm Infants: Systematic Review and Meta-Analysis

**DOI:** 10.3389/fped.2021.681341

**Published:** 2021-06-10

**Authors:** Lingyu Fang, Lianqiang Wu, Shuping Han, Xiaohui Chen, Zhangbin Yu

**Affiliations:** ^1^Division of Neonatology, Quanzhou Women and Children's Hospital, Quanzhou, China; ^2^Department of Pediatrics, Women's Hospital of Nanjing Medical University, Nanjing, China; ^3^Department of Pediatrics, Nanjing Maternity and Child Health Care Hospital, Nanjing, China

**Keywords:** quality improvement, preterm infant, breastfeeding, meta-analysis, breast milk

## Abstract

**Background and Objective:** Due to its numerous health benefits, breast milk (BM) is recommended for preterm infants. Despite such recommendations, the rates of breastfeeding in preterm infants are lower than that in term infants. Quality improvement (QI) bundles increase breastfeeding in preterm infants, but their replication in neonatal intensive care units has had inconsistent outcomes.

**Methods:** We used the Population or Problem, Intervention, Comparison, and Outcomes (PICO) framework to develop our search strategy, and searched MEDLINE, Embase, and the Cochrane Library from inception through January 15, 2021. Studies describing any active QI intervention to increase BM use in preterm infants were included. The primary outcome measure was the rate of any breastfeeding or exclusive mother's own milk (MOM) at discharge or during hospitalization.

**Results:** Sixteen studies were eligible for inclusion and showed an acceptable risk of bias, and included 1 interrupted time series, study 3 controlled before-and-after studies, and 12 uncontrolled before-and-after studies; of these, 3 studies were excluded due to insufficient dichotomous data, 13 were included in the meta-analysis. In the meta-analysis, the rate of any breastfeeding was significantly improved at discharge and during hospitalization after QI [risk ratio (*RR*) = 1.23, 95% confidence interval (CI): 1.14–1.32, *P* < 0.00001 and *RR* = 1.89, 95% CI: 1.09–3.29, *P* = 0.02, respectively]. The rate of exclusive MOM after QI was also significantly increased at discharge (*RR* = 1.51, 95% CI: 1.04–2.18, *P* = 0.03), but not during hospitalization (*RR* = 1.53, 95% CI: 0.78–2.98, *P* = 0.22). However, after sensitivity analysis, the comprehensive results still suggested that QI could significantly improve the rate of exclusive MOM during hospitalization (*RR* = 1.21, 95% CI: 1.08–1.35, *P* = 0.001). Funnel plots and Egger's test indicated publication bias in the rate of any BF at discharge. We corrected publication bias by trim and fill analysis, and corrected *RR* to 1.272, 95% CI: (1.175, 1.369), which was consistent with the results of the initial model.

**Conclusions:** A QI bundle appears to be effective for promoting BM use in preterm infants at discharge or during hospitalization.

## Introduction

The benefits of BM in preterm infants are widely recognized, and include reduction in late-onset sepsis (LOS), necrotizing enterocolitis (NEC), bronchopulmonary dysplasia (BPD), and improved neurodevelopment (1–5). Accordingly, the American Academy of Pediatrics has recommended that all preterm infants should receive human milk; if MOM is unavailable despite significant lactation support, pasteurized donor milk should be used (6). Despite the evidence and policy statement that BM is beneficial for preterm infants, the rate of breastfeeding in preterm infants in neonatal intensive care unit (NICU) as based on region and race/ethnicity remain disparate (7, 8).

Barriers to breastfeeding in preterm infants are NICU encounters, which constitute an environment of mother–infant separation and limited support from lactation consultants (9). QI bundles, intervention methods of evidence-based practices, have been adopted to promote breastfeeding in preterm infants in the NICU, but their replication in the NICU had inconsistent outcomes (10).

Three reviews recently assessed these QI methods for improving breastfeeding in preterm infants in the NICU, but did not quantitatively combine the data and systematically determine the typical effect size of the QI for breastfeeding (11–13). Moreover, newer studies have been published after these reviews. Due to the limitations of the aforementioned reviews, along with continued interest in this subject, we sought to systematically evaluate these studies to increase breastfeeding by preterm infants in the NICU by using QI bundles.

## Methods

This systematic review and meta-analysis followed the Preferred Reporting Items for Systematic Reviews and Meta-Analyses statement (14).

### Search Strategy

We searched Medline, Embase and the Cochrane Library from database inception through January 15, 2021. There were no search limits or restrictions. We identified relevant studies and maximized the search accuracy using the following terms: breastfeeding, MOM, QI, preterm infants. We searched for these terms in the title and abstract.

Supplementary Text 1 describes the detailed search strategy across individual databases. We also searched the references of studies included in the systematic review for other relevant studies.

### Eligibility Criteria

This systematic review aims to determine the effect of QI bundles on breastfeeding by preterm infants in the NICU. We searched for studies using inclusion criteria built on the PICOS framework: (1) Population (P), preterm infants or very-low-birth-weight infants and their mothers were included. (2) Intervention (I), active QI intervention aimed at improving breastfeeding. (3) Comparison (C), infants who did not use the QI bundle as the comparison. (4) Outcome (O), the rate of breastfeeding at discharge or during hospitalization; (5) Study design (S), eligible designs were randomized controlled trials (RCTs), controlled before-and-after study (CBA), uncontrolled before-and-after study (UBA), and interrupted time series study (ITS). Studies that measured the effect of QI bundles on breastfeeding in preterm infants in the NICU were included in the systematic review. Conference abstracts were not accepted. No limitation was applied to the publication language.

### Outcome Measures

If both the historical baseline and an external control group were present, the historical baseline was preferred when comparing clinical outcomes. The primary outcomes were the rates of any breastfeeding or exclusive MOM at discharge or during hospitalization. Any breastfeeding was defined as any amount of MOM, with or without the addition of donor milk, formula, or fortifier. Exclusive MOM was defined as exclusive MOM, with or without fortifier.

The process outcomes included prenatal human milk education, first milk expression within 6 h, any skin-to-skin care in the first month, the number of oropharyngeal therapy doses administered in the first 7 days of life, the proportion of feeding donor human milk, the time at first MOM feeding, the proportion of MOM at initiation of feeds, the proportion of hospital-grade pump use.

The balancing outcomes included NEC and LOS incidence, change in weight gain during hospitalization, length of stay, feeding intolerance incidence, and the time to reach full enteral feeding.

### Study Selection and Data Extraction

Two authors (LF, LW) independently screened titles, abstracts, and full texts for inclusion; in the case of differing opinions, a third author (XC) determined final eligibility. Two authors (LF, LW) independently extracted data from each included study using a standardized data collection form. The following details were extracted from each study: author(s), publication year, setting, location, study design, study duration, target population, primary outcomes, process outcomes, balancing outcomes, intervention items included in the QI bundle. If the abstracted data differed between the two authors, resolution was achieved through discussion or discussion with a third author (ZY). We contacted the corresponding authors when data on the outcomes were missing.

### Quality and Risk of Bias Assessments

Widely used critical evaluation tools fail to take into account the unique characteristics of QI, and existing QI tools [e.g., Standards for Quality Improvement Reporting Excellence (SQUIRE) 2.0 (15)] are intended at guiding publication rather than critical evaluation. The key components of the QI assessment include background, intervention details, and a usage check of the QI process itself. The QI minimum quality criteria set (QI-MQCS) (16) is an effective and reliable assessment tool that can be used for measuring health care QI intervention publications. It includes the following 16 areas or content categories: Organizational Motivation, Intervention Rationale, Intervention Description, Organizational Characteristics, Implementation, Study Design, Comparator, Data Source, Timing, Adherence/Fidelity, Health Outcomes, Organizational Readiness, Penetration/Reach, Sustainability, Spread, and Limitations. Accordingly, we selected the QI-MQCS for the methodological quality assessment of the included studies.

Each QI study was evaluated against the 16 domains, with each domain recording 1 point if it met the minimum criteria, and 0 if it did not. Two reviewers (LF and JZ) applied the tool independently to assess the included studies; discrepancies were resolved by group consensus. According to the study descriptions, we rated these studies as low, medium, and high quality: >10 indicated high quality, 7~10 indicated medium quality, and <7 indicated low quality.

### Statistical Analysis

The QI bundle elements are summarized as frequencies and percentages. To summarize the treatment effect, we report *RR* and 95% CI for dichotomous outcomes. The meta-analysis was conducted using Review Manager 5.3 software. Heterogeneity across studies was tested using *I*^2^ statistics. Possible heterogeneity between studies (*I*^2^ ≥ 50%) was accounted for using a random-effects model, which defaults to the fixed-effects model approach in the absence of heterogeneity. When there was heterogeneity, we sought the source of the heterogeneity and applied sensitivity analysis to observe the effect of each study result on the total effect size. *P* < 0.05 was considered significant. Publication bias was assessed by funnel plots and Egger's test using Stata 12.0.

## Results

**Figure 1:**
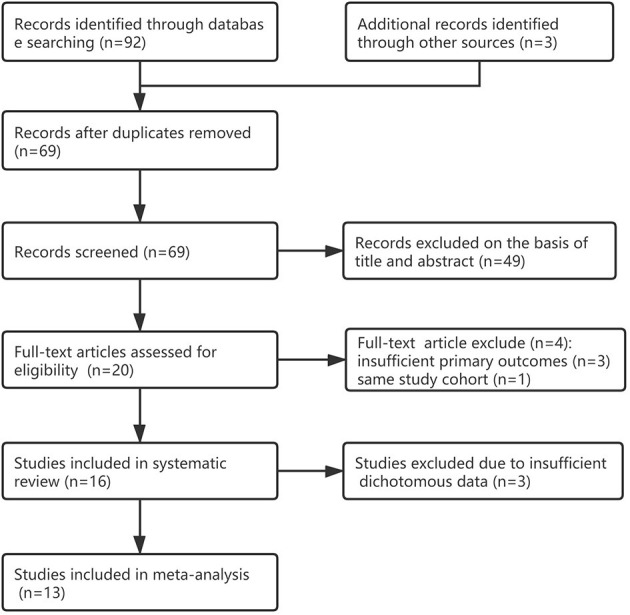
Study selection flow chart.

We identified 95 citations from the database search and other sources as of January 15, 2021. After removing duplicates, the titles/abstracts of 69 articles were screened, and 49 articles were excluded. We reviewed the full text of 20 studies; 16 studies (17–32) were included in systematic review and four studies (33–36) were excluded because of insufficient primary outcomes or same study cohort; 13 studies were included in the meta-analysis for the primary outcome (17–21, 23, 24, 27–32), another 3 studies were excluded due to insufficient dichotomous data. Figure 1 shows the detailed flow chart. One study (36) was excluded because it was within the greater Neonatal QI Collaborative of Massachusetts human milk collaborative (26), three other studies (33–35) were excluded because of insufficient primary outcomes (see Supplementary Table 1).

### Study Characteristics

Most studies were UBAs (12/16), three studies were CBAs, and one was an ITS; Table 1 shows the characteristics of the included studies. The included 16 studies were from the USA (*n* = 8), China (*n* = 3), UK (*n* = 1), Italy (*n* = 1), Canada (*n* = 1), India (*n* = 1), and Spain (*n* = 1). Duration of cohort study was between 2005 and 2019. Most QI (13/16) were from single centers, and the other three were multi-center. The sample sizes ranged from 37 to 33,172 (median = 376 infants). Six reports used gestational age as an inclusion criterion, which ranged from 22 to 37 weeks; 11 used birth weight as an inclusion criterion, including infants under 1,500 g.

**Table 1:** Characteristic of included studies.

**References**	**QI institution(s)**	**Duration**	**Size**	**GA/BW**	**Study design**	**Primary outcome**
Lee et al. (17)	USA, 99 NICU	2008-2011	9,213	BW 401–1,500 g or GA 22–29 w	CBA	(1)
Battersby et al. (18)	UK, 161 NICUs	2009-2012	33,172	GA < 33w	ITS	(1), (2)
Giannì et al. (19)	Italy, 1 NICU	2008-2009 vs. 2011	232	BW ≤ 1,500 g	UBA	(1)
Murphy et al. (20)	USA, 1 NICU	2012-2013	105	BW < 1,500 g	UBA	(1), (2), (3), (4)
Alshaikh et al. (21)	Canada,1 NICU	2009-2012	443	GA < 32 w	UBA	(1), (4)
Dereddy et al. (22)	USA, 1 NICU	2007-2012	1,488	BW < 1,500 g	UBA	(3)
Fugate et al. (23)	USA, 1 NICU	2009-2012	224	BW < 1,500 g	CBA	(1)
Bixby et al. (24)	USA, 1 NICU	2005-2011	309	BW < 1,500 g	UBA	(1)
Liu et al. (25)	China, 1 NICU	2014-2016	9,298	BW < 1,500 g	UBA	(3), (4)
Parker et al. (26)	USA, 9 NICUs	2015-2017	1,670	BW < 1,500 g	UBA	(1), (2)
Bagga et al. (27)	India, 1 NICU	2018-2019	97	GA < 34 w	UBA	(1)
Porta et al. (28)	Spain, 1 NICU	2018-2019	37	BW < 1,500 g	UBA	(1), (2)
Ward et al. (29)	USA, 1 NICU	2006-2016	1,077	BW < 1,500 g	CBA	(1)
Wetzel et al. (30)	USA, 1 NICU	2018	56	GA < 30 w	UBA	(1)
Zhou et al. (31)	China, 1 NICU	2014-2016	488	BW < 1,500 g	UBA	(3), (4)
Yu et al. (32)	China, 1 NICU	2017-2018	70	GA < 37 w	UBA	(3), (4)

*(1) Proportion of infants receiving any breastfeeding at discharge; (2) proportion of infants receiving exclusive MOM at discharge; (3) proportion of infants receiving any breastfeeding during hospitalization; (4) proportion of infants receiving exclusive MOM during hospitalization*.

*GA, gestation age; BW, birth weight*.

### Risk of Bias Assessment

Evaluation of the eligible studies using the QI-MQCS revealed that all studies had scores between 8 and 15: two studies were medium-quality and 14 were high-quality (see Supplementary Table 2). Most studies lacked information on the proportion of all eligible units who actually participated (Penetration/Reach: 10/16), or did not describe the sustainability or the potential for sustainability (Sustainability: 9/16), or did not name the study design (Study design: 9/16). All articles met the minimum quality criteria for five of the 16 areas, i.e., organizational motivation, intervention description, implementation, data sources, and timing.

### Bundled Elements

Table 2 shows a total of 12 interventions included in the QI bundle. The most common professional elements were a multidisciplinary expert team developing evidence-based interventions, education of hospital staff (16/16); and parental education (16/16). The elements of early initiation of milk expression included: increased availability of pumps (14/16); early initiation of human milk expression (14/16); and oropharyngeal administration of colostrum (6/16). The elements of maintenance of lactation included: lactation consultant tracking of visits or phone calls (14/16); skin-to-skin care (8/16); non-nutritive sucking (6/16); human milk management (14/16). Only five studies reported standardized enteral feeding guidelines. Other elements included preparation for discharge (8/16) and post-discharge lactation support and follow-up care (7/16).

**Table 2:** Interventions included in the QI bundle.

**References**	**(1)**	**(2)**	**(3)**	**(4)**	**(5)**	**(6)**	**(7)**	**(8)**	**(9)**	**(10)**	**(11)**	**(12)**
Lee et al. (17)	**+**	**+**	**+**	**+**	**+**	**+**	**+**	**+**	**+**	**+**	**+**	**+**
Battersby et al. (18)	**+**	**+**	**+**	**+**		**+**			**+**	**+**	**+**	
Gianni et al. (19)	**+**	**+**	**+**	**+**		**+**	**+**		**+**	**+**	**+**	
Murphy et al. (20)	**+**	**+**		**+**		**+**						
Alshaikh et al. (21)	**+**	**+**		**+**	**+**	**+**			**+**			
Dereddy et al. (22)	**+**	**+**	**+**			**+**			**+**			
Fugate et al. (23)	**+**	**+**	**+**	**+**	**+**	**+**	**+**	**+**	**+**		**+**	**+**
Bixby et al. (24)	**+**	**+**	**+**	**+**		**+**	**+**	**+**	**+**		**+**	
Liu et al. (25)	**+**	**+**	**+**	**+**	**+**	**+**	**+**	**+**	**+**	**+**	**+**	
Parker et al. (26)	**+**	**+**	**+**	**+**	**+**	**+**	**+**	**+**	**+**	**+**	**+**	**+**
Bagga et al. (27)	**+**	**+**	**+**	**+**		**+**	**+**	**+**	**+**			**+**
Porta et al. (28)	**+**	**+**	**+**	**+**					**+**			
Ward et al. (29)	**+**	**+**	**+**	**+**		**+**	**+**		**+**			**+**
Wetzel et al. (30)	**+**	**+**	**+**	**+**	**+**							
Zhou et al. (31)	**+**	**+**	**+**	**+**		**+**			**+**		**+**	**+**
Yu et al. (32)	**+**	**+**	**+**			**+**			**+**			**+**

*(1) Multidisciplinary expert team, development of evidence-based interventions, education of hospital staff; (2) parental education (prenatal consultations and postnatal education); (3) increase availability of pumps; (4) initiate early human milk expression; (5) oropharyngeal administration of colostrum; (6) lactation consultant tracking of visits or phone calls; (7) skin-to-skin care; (8) non-nutritive sucking; (9) human milk management; (10) standardized enteral feeding guideline; (11) preparation for discharge (family-integrated care, transition to direct breastfeeding); (12) post-discharge lactation support and follow-up care*.

### The Primary Outcomes

Three studies (22, 25, 26) did not report sufficient dichotomous data, which we did not include in the meta-analysis. An inner-city hospital implemented a multipronged approach for very-low-birth-weight infants over 5 years, and the rates of any breastfeeding during hospitalization improved gradually from 22% in the July-September 2007 quarter to 88% in the October-December 2012 quarter (*P* < 0.0001) (22). There was a statistically significant increase in the proportion of MOM to human milk during hospitalization following the implementation of a QI bundle (57% vs. 86%) in a Chinese level III NICU (25). However, the Massachusetts statewide QI collaborative in the USA did not improve the rates of any breastfeeding or exclusive MOM at discharge after conducting 69 interventions from January 2015 to December 2017 compared to baseline data from 2011 to 2014 (63.7% vs. 63%, and 46.4% vs. 45%, respectively) (26).

**Figure 2:**
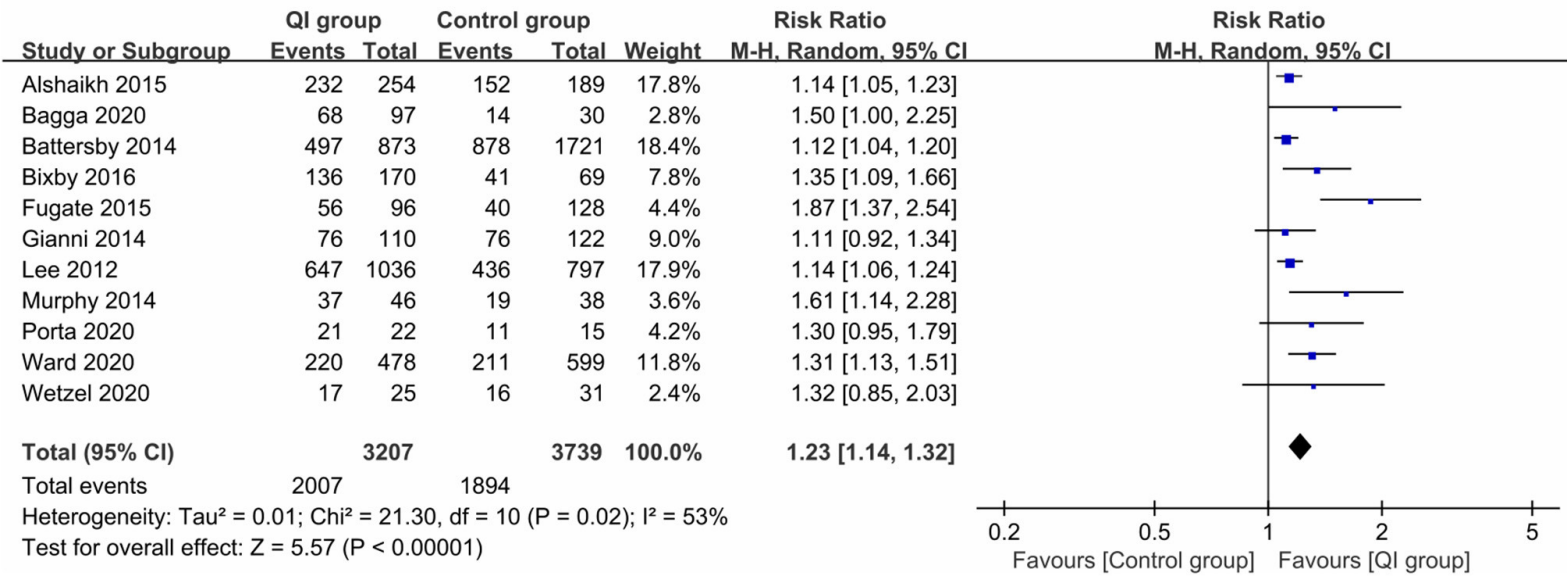
Forest plot from random effects analysis: The rate of any breastfeeding at discharge before-and-after QI.

Eleven studies (17–21, 23, 24, 27–30) reported the rate of any breastfeeding at discharge, involving 3,207 infants in the QI group and 3,739 infants in the control group; the combined results showed a significantly positive relation between QI bundles and the proportion of any breastfeeding (*RR* = 1.23, 95% CI: 1.14–1.32, *P* < 0.00001) (Figure 2). As the heterogeneity between studies was large (*I*^2^ = 53%), we examined the effect of each study on overall risk estimates by excluding one study at a time. When the study by Fugate et al. (23) was removed, the inter-study heterogeneity decreased to 26%, and the pooled RR remained at 1.18 (95% CI: 1.12–1.24, *P* < 0.0001). The heterogeneity may have been due to the difference in the contents of the QI bundles.

**Figure 3:**
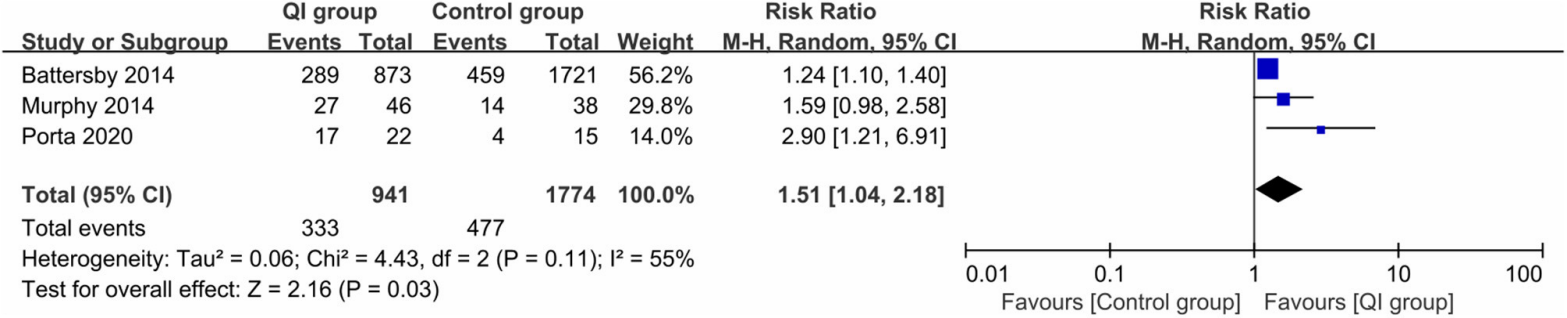
Forest plot from random effects analysis: The rate of exclusive MOM at discharge before-and-after QI.

Three studies (2,715 infants) (18, 20, 28) reported the rate of exclusive MOM at discharge. The heterogeneity between studies was large (*I*^2^ = 55%). The meta-analysis revealed a statistically significant increase in the rate of exclusive MOM at discharge following the introduction of QI bundles (*RR* = 1.51, 95% CI: 1.04–2.18, *P* = 0.03) (Figure 3). The sample size of the study by Porta et al. (28) was too small (*n* = 37), and may have been a source of heterogeneity. Excluding that study (28) led to homogeneity among the studies (*P* = 0.32, *I*^2^ = 0%), and the meta-analysis result remained stable (*RR* = 1.26, *P* = 0.0001).

**Figure 4:**
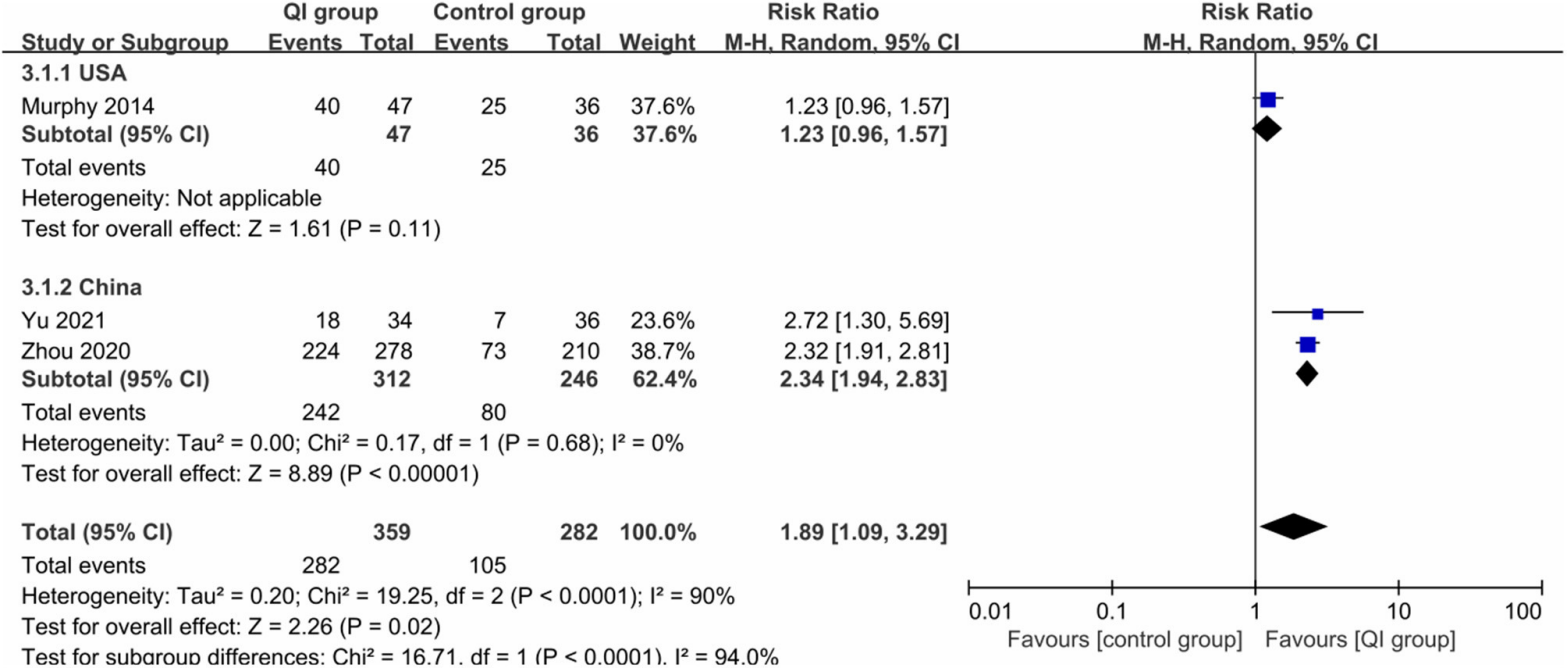
Forest plot from random effects and subgroup analysis: The rate of any breastfeeding during hospitalization before-and-after QI.

Three studies (641 infants) (20, 31, 32) reported the rate of any breastfeeding during hospitalization, with high heterogeneity (*I*^2^ = 90%), and QI improved the rate of any breastfeeding during hospitalization (*RR* = 1.89, 95% CI: 1.09–3.29, *P* = 0.02) (Figure 4). Subgroup analysis by country showed that heterogeneity was low among the Chinese studies (*I*^2^ = 0%), and the rate was significantly improved after QI (*RR* = 2.34, 95% CI: 1.94–2.83, *P* < 0.00001), which may be related to the higher compliance and acceptance of the participants.

**Figure 5:**
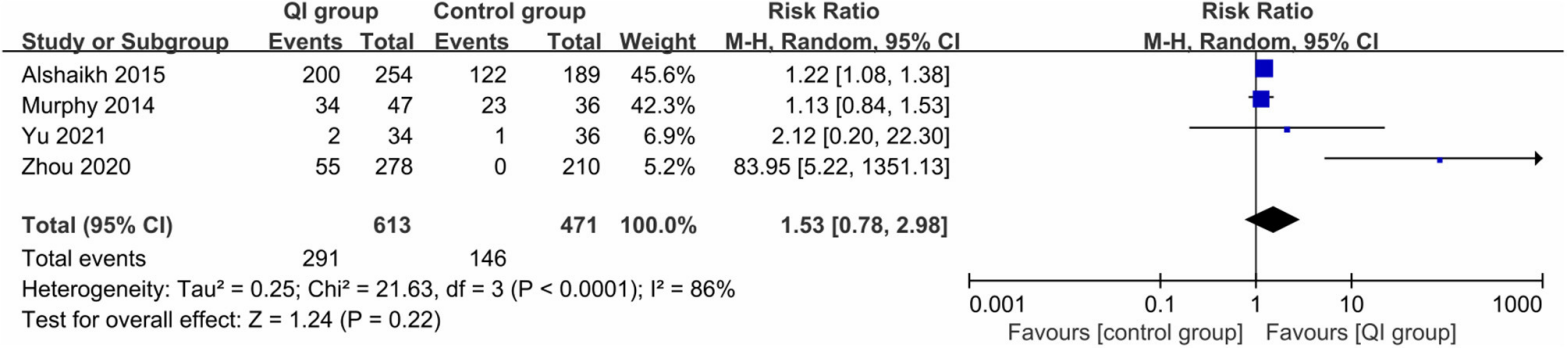
Forest plot from random effects analysis: The rate of exclusive MOM during hospitalization before-and-after QI.

Four studies (1,084 infants) (20, 21, 31, 32) reported the rate of exclusive MOM during hospitalization, for which large heterogeneity was found (*I*^2^ = 86%), and QI did not make a significant difference between the two groups (*RR* = 1.53, 95% CI: 0.78–2.98, *P* = 0.22) (Figure 5). In the study measurements of Zhou et al. (31), there was a statistical value of 0; when we removed this study, the heterogeneity was dramatically reduced (*I*^2^ = 0%), indicating that it was the source of heterogeneity, and the comprehensive results suggested that QI could still significantly increase the rate of exclusive MOM during hospitalization (*RR* = 1.21, 95% CI: 1.08–1.35, *P* = 0.001).

### The Process Outcomes

Three studies (17, 22, 24) did not report the major process outcomes (Table 3). Two different studies reported the process outcomes of prenatal human milk education (26, 32), first milk expression within 6 h (23, 26), and any MOM at initiation of feeds separately (21, 23), which were all improved significantly during the QI interventions. One study reported that QI improved any skin-to-skin care in the first month (26), oropharyngeal therapy doses administered in the first 7 days of life (30), and the rate of using hospital-grade pumps (23), respectively, but another study (25) showed a decline in the proportion of feeding donor human milk and no difference in the time of first MOM feeding. Twelve studies reported other different process outcomes (see Supplementary Table 3).

**Table 3:** Process outcomes and balancing outcomes included in the QI bundle.

**References**	**Process outcomes**									**Balancing outcomes**								
	**(1)**	**(2)**	**(3)**	**(4)**	**(5)**	**(6)**	**(7)**	**(8)**	**(9)**		**(10)**	**(11)**	**(12)**	**(13)**	**(14)**	**(15)**	**(16)**	**(17)**
Lee et al. (17)	Not stated		↓				#											
Battersby et al. (18)									↑		Not stated							
Gianni et al. (19)									#				↓					#
Murphy et al. (20)									#						#			
Alshaikh et al. (21)							↑		↑		↓	#			#		#	#
Dereddy et al. (22)	Not stated		Not stated															
Fugate et al. (23)		↑					↑	↑	#				#		#			
Bixby et al. (24)	Not stated		Not stated															
Liu et al. (25)					↓	#			↓					#		↓	↓	
Parker et al. (26)	**↑**	↑	↑								#	#		#	#			
Bagga et al. (27)									↑		#	#			#		#	#
Porta et al. (28)									#		Not stated							
Ward et al. (29)									↓						#			
Wetzel et al. (30)				↑							#	#						#
Zhou et al. (31)									↑		#	#			#		#	#
Yu et al. (32)	↑								↑		Not stated							

*(1) Prenatal human milk education, (2) first milk expression within 6 h, (3) any skin-to-skin care in the first month, (4) the number of oropharyngeal therapy doses administered in the first 7 days of life, (5) the proportion of feeding donor human milk, (6) the time to first MOM feeding, (7) any MOM at initiation of feeds, (8) the rate of using hospital-grade pumps, (9) other process outcomes, (10) NEC, (11) sepsis, (12) EUGR, (13) change in weight gain during hospitalization, (14) length of stay, (15) feeding intolerance, (16) the time to reaching full enteral feeding, (17) other balancing outcomes*.

***↓:** Decrease; **↑:** increase; **#:** no statistical significance*.

### The Balancing Outcomes

Five studies did not report the balancing outcomes (18, 22, 24, 28, 32) (Table 3). Two studies (17, 21) showed that the risk of NEC decreased significantly after implementation of the QI bundle, but four studies (26, 27, 30, 31) showed no statistical differences. Five studies (21, 26, 27, 30, 31) showed that QI did not affect the LOS rate, and three studies (23, 25, 26) showed that the QI bundle did not affect weight gain during hospitalization or risk of extrauterine growth retardation (EUGR). There were no differences in length of stay in eight studies (17, 20, 21, 23, 26, 28, 29, 31). Three studies (21, 27, 31) reported that the time of reaching full enteral feeding was not statistically different, other than the study by Liu et al. (25). Only one study reported decreased risk of feeding intolerance (25). Five studies consistently showed that QI bundle did not influence other balancing outcomes (see Supplementary Table 3).

### Publication Bias

**Figure 6:**
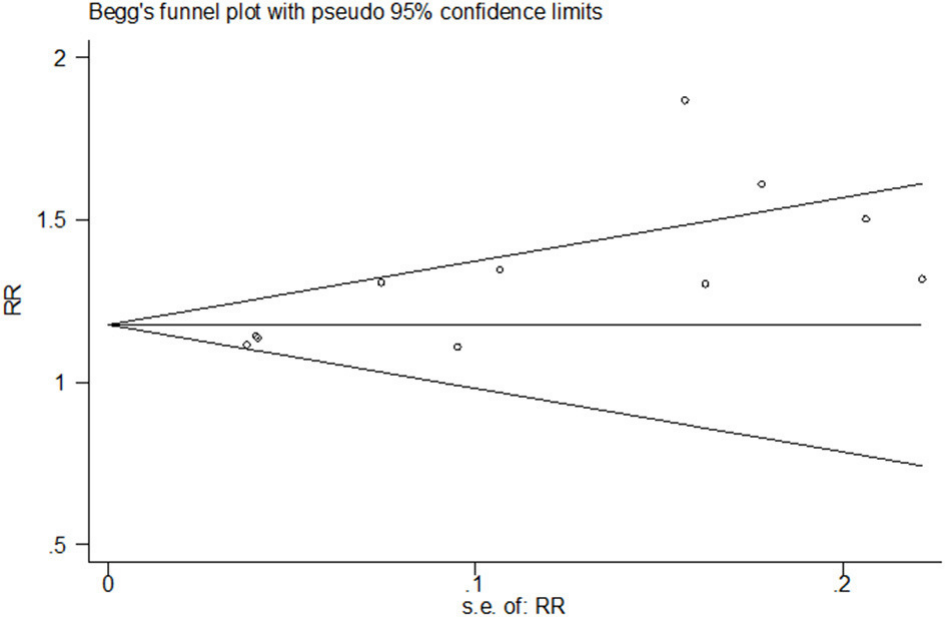
Funnel plot of publication bias for the rate of any breastfeeding at discharge.

Both funnel plots and Egger's test of the intercept indicated the presence of publication bias. Visual examination of the funnel plot showed that it was asymmetric (Figure 6) and Egger's test showed statistical significance (*t* = 3.77, *P* = 0.004).

**Figure 7:**
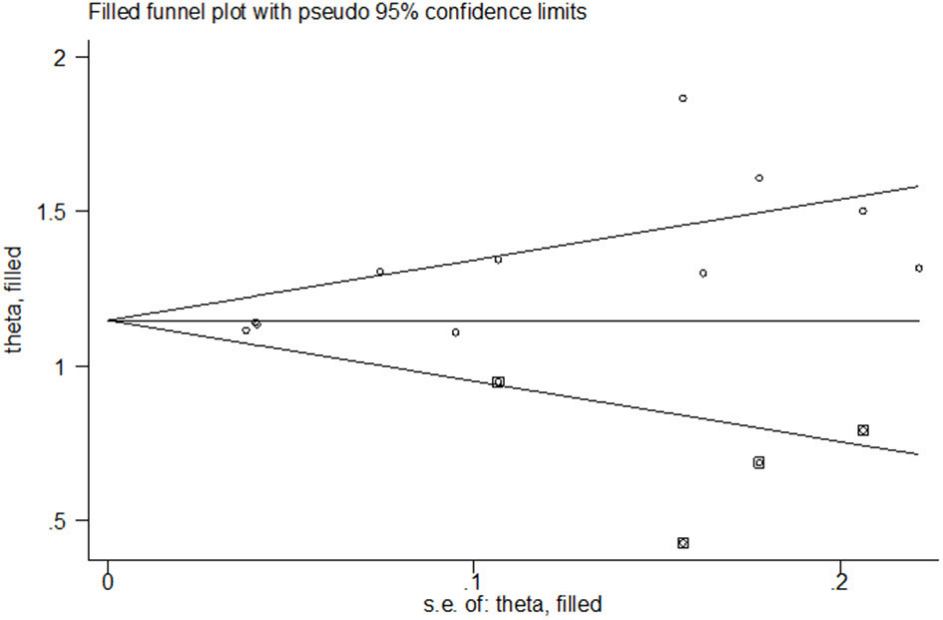
Funnel plot of publication bias for the rate of any breastfeeding at discharge after correction.

To correct the publication bias, we applied a trim and fill analysis in the random-effects model (Figure 7) by adding four articles; the corrected *RR* was 1.272, 95% CI: (1.175, 1.369). The result showed no significant difference in the estimation of breastfeeding rate between the initial model and the trim-and-fill model.

## Discussion

This systematic review suggests that the QI bundle is associated with a statistically significant increase in the rate of any breastfeeding and MOM feeding in preterm infants at discharge or during hospitalization. The QI bundle included 12 interventions, all involved in establishing local evidence-based studies, with varying levels of process change incorporated. Although the meta-analysis revealed that there was considerable heterogeneity in the rates of breastfeeding and MOM in at discharge or during hospitalization, the results were stable when using geographical subgroup analysis or sensitivity analysis to exclude sources of heterogeneity, such as studies with too-small sample sizes (28) or zero outcome data (31).

QI is aimed at improving the quality of health care, and involves investing a great deal of manpower and resources. Effective, reliable key assessment tools promote the impact of QI by helping stakeholders identify higher-quality research. The SQUIRE team developed detailed reporting guidelines for the publication of QI interventions to help authors describe QI interventions (15). It aims to ensure that readers can appraise and understand their interventions and evaluations by identifying the details of their reports for authors. The QI-MQCS is intended to serve as a resource for reviewers to help synthesize the large amount of evidence available for QI interventions and to provide a framework for critical evaluation (16).

In the present study, assessment of the included studies using QI-MQCS revealed that 14 studies (14/16) were of high quality. Even though 13 studies (13/16) reported improvements, the widespread challenges, with low adherence to key methodological items in the individual projects, posed a challenge to the legitimacy of QI interventions. The present review indicates that further study is needed for improving the QI methodology.

There are also a number of effective and reliable key assessment tools that promote the impact of QI by helping stakeholders identify higher-quality research. The concept of rapid cycle improvement, or the Plan-Do-Study-Act cycle, first appeared in papers published in 2009–2011 (37, 38). Since 2015, the reference about improved model, as well as tools such as key drive diagrams and Pareto charts have appeared in publications. However, only one study has described the promotion of QI in BM using the key driver diagram and the Plan-Do-Study-Act cycle (27).

Maintenance of lactation is usually measured by whether the infant is still partially or exclusively breastfed when discharged from the NICU, so most of the studies (11/16) used any breastfeeding or MOM at discharge. However, there remain outcome measures that are not consistently shown or that are ignored. First, the outcome measure does not capture the timing of human milk initiation. Several studies have shown that early hand expression and/or breastfeeding is associated with increased MOM production (39, 40), which increases when initiation occurs within 6 h after delivery (40). Mothers who start breastfeeding within 1 h after delivery produce more milk than those who start between 1 and 6 h after delivery (39). Furthermore, the benefits of colostrum are self-evident. Therefore, the time of breastfeeding initiation should be measured as an important outcome.

Second, evidence-based quality indicators targeting high doses of breastfeeding should be emphasized. A prospective cohort study (41) found that a considerable proportion (60.7%) of very-low-birth-weight infants without breastfeeding at discharge received high-dose breastfeeding within 14 and 28 days after birth; some had received higher amounts of MOM than infants who received exclusive and partial MOM at discharge. Early high-dose breastfeeding can significantly reduce the risk of a variety of morbidities in very-low-birth-weight patients, including LOS, NEC, and BPD, and reduce the associated costs (42–44). This emphasizes the need to collect the dose of BM (in mL/kg/d or as a proportion of total enteral feeding).

The main process measures involved in parental education, early initiation of milk expression and maintenance of lactation were reported only 11 times (8.6%). Three studies did not report the process measure. Accordingly, future studies should measure the main process outcomes, which could be used to assess the association between the process measures and improved primary outcomes.

Preterm infants fed unfortified BM are at increased risk for slow growth during hospitalization; change in weight gain during hospitalization or the percentage of infants with extrauterine growth retardation were often chosen as the balancing measure. Four studies (25%) used it as the balancing measure. The benefits of BM (NEC, LOS, feeding intolerance, length of stay, time to reach full enteral feeding) were chosen as the balancing measure, which were used 24 times. Future studies should measure the main balancing outcomes, which could be used to assess unintended consequences (balancing outcomes) such as unexpected benefits, and problems associated with the intervention.

There was variability within the QI bundles, although the most common elements included a multidisciplinary expert team, development of evidence-based interventions, education of hospital staff; parental education; increased availability of pumps; initiating early human milk expression; lactation consultant tracking of visits or phone calls; and human milk management. Other bundles (oropharyngeal administration of colostrum; skin-to-skin care; non-nutritive sucking; standardized enteral feeding guidelines) potentially affecting breastfeeding was not frequently reported. We could not assess the association between the specific bundled elements and increased breastfeeding.

The systematic review establishes the effect of a QI bundle in promoting BM use by preterm infants in the NICU. Individual units that have identified low proportion of BM use in the NICU as an issue will find our results useful, as we have compiled the results of 16 relevant studies. This allows the unit to implement the QI bundle and improve BM use, allowing it to alter and adjust their interventions and ensure the best possible response to its implementation.

An important limitation of this review is the potential for publication bias. QIs that result in a change are more likely to be published than QIs that do not result in an improvement. At present, there is no formal method for evaluating QI publication bias; these methods will make important contributions to the research of QI in future systems. While there may be valuable lessons to be learned from the unpublished QI on breastfeeding, the lessons described in the published projects described above remain useful in the quest to increase breastfeeding rates and volumes in the NICU. Second, due to the sample size, the characteristics of the interventions, and the numerical value of the results, the study has high heterogeneity. Third, the included studies did not report some process outcomes, it is not clear what bundle elements are most effective in the NICU. Future research should focus on determining the processes that promote the effective implementation of promoting breastfeeding, and which bundle elements represent essential components.

## Conclusion

There is now substantial evidence suggesting that implementing a QI bundle appears to be effective in promoting any breastfeeding and MOM in preterm infants at discharge. However, some process outcomes were not reported, and it is not clear what bundle elements are most effective in the NICU. Future research should focus on determining what processes promote the effective implementation of promoting any breastfeeding and MOM, and which bundle elements represent essential components.

## Data Availability Statement

The datasets presented in this study can be found in online repositories. The names of the repository/repositories and accession number(s) can be found in the article/Supplementary Material.

## Author Contributions

ZY and SH conceived the review. LF and LW searched the literature and extracted the data. LF, LW, and XC assessed the study quality. LF and LW performed the meta-analysis. ZY, SH, and XC interpreted the data. LF and LW drafted the original manuscript. All authors were involved in critical revision of the article and approved the final version for publication.

## Conflict of Interest

The authors declare that the research was conducted in the absence of any commercial or financial relationships that could be construed as a potential conflict of interest.
