# Supplementary material for: Quality Improvement to Increase Breastfeeding in Preterm Infants: Systematic Review and Meta-Analysis
Source: Front Pediatr. 2021 Jun 10;9:681341. doi: 10.3389/fped.2021.681341 (PMC8222601; doi:10.3389/fped.2021.681341)
Supplement: Supplementary file 1 [file Table_1.DOCX]

**S2 Table. Characteristic of Excluded Studies.**

| **Author, Year** | **Location** | **Duration** | **Target population** | **Primary outcome** | **Intervention** |
| --- | --- | --- | --- | --- | --- |
| Ward 2012 [33] | USA | 2006~2010 | BW < 1500 g  (n = 460) | The number of infants receiving at least 500 mL of human milk/kg in their first 14 days of life increased from 50% to 80%. | Staff education, Antenatal consults, staff assistance bundle for mothers in establishing and maintaining their milk supply |
| Thakur 2018 [34] | India | 2015~2016 | BW < 1500 g  (n = 125) | Increasing the mean proportion of expressed breast milk once infant reached a feed volume of 100 mL/kg/day (61.3% vs 82.3%) | Availability of breast pumps, availability of physical help, kangaroo mother care, non-nutritive sucking, daily team huddle |
| Mallikarjuna 2019 [35] | India | Aug-Nov 2015 | Mothers who delivered VLBW infants (n = 31) | Increasing the milk output on day 7 (113.6mL vs 182mL) | Early expression of milk, use of breast pumps, frequent expressions, videos, and regular counseling |
