# Supplementary material for: Quality Improvement to Increase Breastfeeding in Preterm Infants: Systematic Review and Meta-Analysis
Source: Front Pediatr. 2021 Jun 10;9:681341. doi: 10.3389/fped.2021.681341 (PMC8222601; doi:10.3389/fped.2021.681341)
Supplement: Supplementary file 2 [file Table_2.doc]

**Table S2. The risk of bias in each eligible study.** The included studies were evaluated using QI-MQCS.

| Study | D1 | D2 | D3 | D4 | D5 | D6 | D7 | D8 | D9 | D10 | D11 | D12 | D13 | D14 | D15 | D16 | Total | Study Quality |
| --- | --- | --- | --- | --- | --- | --- | --- | --- | --- | --- | --- | --- | --- | --- | --- | --- | --- | --- |
| Lee 2012 [17] | 1 | 1 | 1 | 1 | 1 | 0 | 1 | 1 | 1 | 1 | 1 | 1 | 1 | 1 | 1 | 1 | 15 | High |
| Battersby 2014 [18] | 1 | 1 | 1 | 1 | 1 | 1 | 1 | 1 | 1 | 1 | 0 | 0 | 1 | 0 | 0 | 1 | 11 | High |
| Gianni 2014 [19] | 1 | 1 | 1 | 1 | 1 | 1 | 1 | 1 | 1 | 0 | 1 | 1 | 0 | 0 | 1 | 1 | 13 | High |
| Murphy 2014 [20] | 1 | 0 | 1 | 1 | 1 | 0 | 1 | 1 | 1 | 1 | 0 | 0 | 1 | 0 | 1 | 1 | 11 | High |
| Alshaikh 2015 [21] | 1 | 1 | 1 | 0 | 1 | 1 | 0 | 1 | 1 | 0 | 1 | 0 | 0 | 1 | 1 | 1 | 11 | High |
| Dereddy 2015 [22] | 1 | 0 | 1 | 1 | 1 | 0 | 1 | 1 | 1 | 1 | 1 | 1 | 0 | 1 | 1 | 0 | 12 | High |
| Fugate 2015 [23] | 1 | 0 | 1 | 1 | 1 | 0 | 1 | 1 | 1 | 1 | 1 | 1 | 0 | 1 | 1 | 1 | 13 | High |
| Bixby 2016 [24] | 1 | 1 | 1 | 0 | 1 | 0 | 1 | 1 | 1 | 1 | 0 | 1 | 1 | 1 | 1 | 0 | 12 | High |
| Liu 2016 [25] | 1 | 1 | 1 | 0 | 1 | 0 | 0 | 1 | 1 | 0 | 1 | 0 | 0 | 0 | 1 | 0 | 8 | Medium |
| Parker 2019 [26] | 1 | 1 | 1 | 1 | 1 | 1 | 0 | 1 | 1 | 1 | 0 | 1 | 1 | 1 | 1 | 1 | 14 | High |
| Bagga 2020 [27] | 1 | 1 | 1 | 1 | 1 | 0 | 0 | 1 | 1 | 1 | 1 | 1 | 0 | 0 | 1 | 1 | 12 | High |
| Porta 2020 [28] | 1 | 1 | 1 | 0 | 1 | 1 | 0 | 1 | 1 | 0 | 0 | 0 | 0 | 0 | 1 | 1 | 9 | Medium |
| Ward 2020 [29] | 1 | 1 | 1 | 1 | 1 | 1 | 0 | 1 | 1 | 1 | 0 | 1 | 1 | 1 | 1 | 1 | 14 | High |
| Wetzel 2020 [30] | 1 | 0 | 1 | 1 | 1 | 0 | 0 | 1 | 1 | 1 | 1 | 1 | 0 | 0 | 1 | 1 | 11 | High |
| Zhou 2020 [31] | 1 | 1 | 1 | 1 | 1 | 1 | 0 | 1 | 1 | 1 | 1 | 1 | 0 | 0 | 1 | 1 | 13 | High |
| Yu 2021 [32] | 1 | 1 | 1 | 1 | 1 | 0 | 0 | 1 | 1 | 1 | 0 | 1 | 0 | 0 | 1 | 1 | 11 | High |

QI-MQCS: minimum quality standards for quality improvement; D1: Organizational Motivation; D2: Intervention Rationale; D3: Intervention Description; D4: Organizational Characteristics; D5: Implementation; D6: Study Design; D7: Comparator Description; D8: Data Sources; D9: Timing; D10: Adherence/Fidelity; D11: Health Outcomes; D12: Organizational Readiness; D13: Penetration/Reach; D14: Sustainability; D15: Spread; D16: Limitations.

Three grades of quality were used for each study, based on the score achieved in the critical appraisals: > 10 was high quality, 7~10 was medium quality, and ﹤7 was low quality.
