# Supplementary material for: Quality Improvement to Increase Breastfeeding in Preterm Infants: Systematic Review and Meta-Analysis
Source: Front Pediatr. 2021 Jun 10;9:681341. doi: 10.3389/fped.2021.681341 (PMC8222601; doi:10.3389/fped.2021.681341)
Supplement: Supplementary file 3 [file Table_3.DOCX]

**S4 Table. The characteristics of the process outcomes and balancing outcomes.**

| **Study** | **Process outcomes** | **balancing outcomes** |
| --- | --- | --- |
| Lee 2012 [17] | Not stated | Necrotizing enterocolitis rates decreased from baseline (7.0%) to intervention period (4.3%) to sustainability period (2.4%). Mean lengths of stay increased between baseline and implementation phases but returned to baseline levels during the sustainability phase (67.5d vs 71.3d vs 66.8d). |
| Battersby 2014 [18] | The change of percentage of care days with any maternal breast milk in trend from preintroduction to postintroduction did show a significant improvement (difference in trends 0.5% (0.04, 1.0), p=0.03). | Not stated. |
| Gianni 2014 [19] | 81% of infants in the historical group were fed human milk at full enteral feeding attainment, whereas in the intervention group was 76% (p=0.37). At discharge the percentage of infants fed breast milk >50% of total milk intake was higher in the historical group than in the intervention one (70% vs 52%, p = 0.03). However, when controlling for multiple pregnancies, this difference could not be detected any longer. | The rate of extrauterine growth retardation at discharge decreased in the intervention group than in the historical group (56% vs. 78%, p=0.001). But the rates of chronic lung disease and any severe infection were not different. |
| Murphy 2014 [20] | The median first maternal milk expression decreased from 9 (25th, 75th percentile; 6, 16) hours to 6 (5, 11) hours after implementation (P=0.06). The proportion of infants receiving exclusive maternal breast milk at 28 days and at discharge was 64% and 74%, respectively (P = 0.40). | There was no significant difference in length of stay (55d vs. 54d). |
| Alshaikh 2015 [21] | The rate of exclusive human milk at starting feed and exclusive human milk at 40 ml/kg/d was significantly increased (60.8% vs 73.6%, 64.5 vs 78.8%), but the rate of exclusive human milk at full feed was no different. | The risk of NEC and sepsis decreased significantly in the sustain period (8.9% vs 4.7%, 17.4% vs 13.4%), and the riks of severe IVH, PDA, BPD, ROP was no different.  Time to reach full feed and length of stay remained similar between the baseline and sustain periods. |
| Dereddy 2015 [22] | Not stated | Not stated |
| Fugate 2015 [23] | There was an increase in the Press Ganey mean satisfaction score for “nurses’ support of mother’s efforts to breastfeed” from 75 to 93.8 (p=0.188), Significant improvements were achieved in the percentages of mothers expressing their milk within 6 hours of delivery, infants receiving MOM at initiation of feeds, and mothers with a hospital-grade pump at discharge. | The percentage of infants less than the third percentile on the Fenton  Growth Chart at initial discharge increased after implementation of the QI methodology (16.4% vs 23.8%, p = 0.161). The length of stay remained similar between the baseline and sustain periods (74.2d vs 67.9d, p=0.241). |
| Bixby 2016 [24] | Not stated | Not stated |
| Liu 2016 [25] | The time of first MOM feeding decreased from 73h to 67h (p=0.139), the percentage and amount of feeding donor human milk during hospitalization significantly decreased (43% vs 14%, 1204ml vs 153ml, respectively）. | The incidence of feeding intolerance decreased from 47.5% to 35.3%,p=0.032; the time of reaching full enteral feeding decreased after QI (19d vs. 18d, P=0.012), there was no significant change in the weight gain during hospitalization (10.32 g/kg/d vs. 10.71 g/kg/d). |
| Parker 2019 [26] | Prenatal human milk education increased from 57.6% to 76.6%, first milk expression within 6 hours increased from 36.6% to 57.1%, and any skin-to-skin care in the first month increased from 31.2% to 39.0%. | There were no difference in the incidence of any necrotizing enterocolitis, late-onset sepsis, weight for gestational age z score  change (growth), or length of stay. |
| Bagga 2020 [27] | The proportion of preterm neonates who received MOM within first 24 hours improved significantly (p<0.0001) from 24% to 83% in the initiation phase and remained stable (90%) in the continuation phase. The amount of MOM received on day 1, day 3, day 7 increased significantly (p<0.05). | The number of babies requiring TPN was significantly lesser during the continuation phase, 54.6% vs 26.7%, p=0.02). Other outcomes such as time to reach full enteral feeds, time to regain birth weight, rates of sepsis, NEC, BPD, hs-PDA, and duration of hospital stay were comparable in both phases. |
| Porta 2020 [28] | The mean time of first pumping after delivery and the time of first colostrum was comparable before and after QI (p﹥0.05), the daily milk average volume obtained by day 14 was significantly higher after QI, 510ml versus 230 mL (p < 0.001). | Not stated |
| Ward 2020 [29] | The median time to pump initiation after intervention decreased from 11 to 5 hours, p = 0.0001. | There was no difference in length of stay（53d vs 53.5d）. |
| Wetzel 2020 [30] | There was a 40% increase (from 24% to 64%) in the number of OPT doses administered in the first 7 days of life. | There was no significant change in the outcomes of NEC, LOS, or  Death. |
| Zhou 2020 [31] | High-volume mother's milk and colostrum feeding rates increased significantly from baseline after the implementation of our mother's milk promotion program(22.8% vs 61.6%, 1.4% vs 21.5%,p<0.01). | The intervention group decreased incidence of NEC needing surgery (7.62% vs. 3.24%, OR 0.32, 95% CI: 0.14–0.76). After adjusting for confounding factors, there were no significant differences in rates of mortality, NEC, infection, PVL/IVH, ROP, BPD, days to full feeds and in NICU. |
| Yu 2021 [32] | The frequec1y of nurses' health education about breastfeeding for mothers of premature newborns significantly increased (p=0.015). The score of nurses’theoretical scores and maternal lactation skills significantly increased after the intervention respectively (69.8 vs 96.53, 56.5 vs 92.1). Maternal lactation volume was significantly greater than the at the preintervention audit in the first, second, and third 24 hours after birth (p<0.01). | Not stated. |
