# Supplementary material for: Quality Improvement to Increase Breastfeeding in Preterm Infants: Systematic Review and Meta-Analysis
Source: Front Pediatr. 2021 Jun 10;9:681341. doi: 10.3389/fped.2021.681341 (PMC8222601; doi:10.3389/fped.2021.681341)
Supplement: Supplementary file 4 [file Table_4.DOCX]

**S1 Text. Search strategy ( from database inception to January 15, 2021)**

**Medline Search Strategy via Pubmed**

#1 “Breast Milk”[tiab] OR breastmilk[tiab] OR “Human Milk”[tiab] OR “Breast Feeding”[tiab] OR breastfeeding [tiab] OR “mother's Milk”[tiab] OR “Mother's Own Milk”[tiab] 58120

#2 “Quality Improvement*” [tiab] 41711

#3 ”premature infant*”[tiab] OR ”prematurity infant*”[tiab] OR ”preterm infant*”[tiab] OR “low birth weight”[tiab] OR "low birthweight"[tiab] OR VLBW[tiab] OR LBW[tiab] 75079

#1 AND #2 AND #3 56

**Embase Search Strategy**

#1 'mothers milk':ti,ab,kw OR 'breast milk':ti,ab,kw OR 'breast feeding':ti,ab,kw OR 'mothers own milk':ti,ab,kw 32131

#2 'quality improvement':ti,ab,kw 63557

#3 prematurity:ti,ab,kw OR 'preterm infants':ti,ab,kw OR 'preterm infant':ti,ab,kw OR 'low birth weight':ti,ab,kw 93,175

#1 AND #2 AND #3 25

**Cochrane Library Search Strategy**

#1 MeSH descriptor: [Breast Feeding] explode all trees 1880

#2 MeSH descriptor: [Milk, Human] explode all trees 1013

#3 MeSH descriptor: [Breast Milk Expression] explode all trees 29

#4 #1 OR #2 OR #3 2553

#5 Quality Improvement 38175

#6 (infant OR infants OR infantile OR infancy OR newbORn* OR "new born" OR "new borns" OR "newly born" OR neonat* OR baby* OR babies OR premature OR prematures OR prematurity OR preterm OR preterms OR "pre term" OR premies OR "low birth weight" OR "low birthweight" OR VLBW OR LBW OR ELBW OR NICU):ti,ab,kw 87044

#7 #4 AND #5 AND #6 23 reviews and 11 trials
